# Genital *Dirofilaria*: A Comprehensive Review of Clinical Presentations, Diagnosis and Management

**DOI:** 10.1007/s11686-026-01271-8

**Published:** 2026-05-19

**Authors:** Alessandro Franzò, Agnese Maria Isabella Messina, Roberto Bruno, Barbara Bellocchi, Eugenia Pistarà, Federica Cosentino, Serena Spampinato, Benedetto Maurizio Celesia, Bruno Santi Cacopardo, Giuseppe Nunnari, Andrea Marino

**Affiliations:** 1 Unit of Infectious Diseases, Department of Clinical and Experimental Medicine, ARNAS Garibaldi Hospital, University of Catania, Catania, 95122 Italy; 2 Faculty of Medicine and Surgery Department of Experimental and Clinical Medicine, University of Catania, Catania, 95122 Italy

**Keywords:** Dirofilariasis, *Dirofilaria repens*, Genital masses, Subcutaneous nodules, Vector-borne diseases, Zoonosis

## Abstract

**Introduction:**

Human dirofilariasis, an emerging mosquito-borne zoonosis, is increasing, particularly in Europe. While subcutaneous presentations are the most common infections, genital presentations are rare and can pose significant diagnostic challenges.

**Purpose:**

This review aims to provide an updated synthesis of all detailed published cases of genital dirofilariasis analyzing clinical characteristics, diagnostic methods, treatment strategies, and epidemiological implications to enhance clinical awareness and management.

**Methods:**

A comprehensive literature review was conducted to identify cases of *Dirofilaria* species with genital involvement in databases such as PubMed, Scopus, Web of Science and Google Scholar and through manual searches.

**Results:**

Genital dirofilariasis is a rare condition, with 70 detailed documented cases identified in this review, representing the largest collection published to date. *Dirofilaria repens* is the predominant cause. Clinical presentation is often non-specific mimicking common or threatening conditions such as orchiepididymitis, spermatic cord torsion or undiagnosed nodule. The condition affects all ages, with a notable 40% of cases occurring in the pediatric population, particularly in Sri Lanka. Definitive diagnosis typically requires surgical excision and expert histopathological examination, supported by molecular techniques for species identification, if available and certificated. Imaging, mainly ultrasound, may reveal the highly suggestive “worm-in-sac sign”. Minimally invasive excisional surgery is generally curative and post-operative antiparasitic therapy is usually not required after complete excision.

**Conclusions:**

Genital dirofilariasis should be included in the differential diagnosis of unexplained genital nodules or masses, especially in endemic areas or in patients with a history of travel to such regions. The diagnostic and therapeutic approach proposed here helps in identifying and managing these peculiar parasitic infections. A multidisciplinary approach is essential to avoid misdiagnosis and inappropriate invasive interventions.

## Introduction

*Dirofilaria* belongs to the superfamily Filarioidea and represents a significant example of mosquito-transmitted nematodes with a zoonotic potential [1]. Amongst the several species included within the genus *Dirofilaria*,* Dirofilaria immitis* and *Dirofiliaria repens* are the most important members [2]. These parasites are transmitted through the bites of infected mosquitoes, mainly from the *Aedes*, *Culex*, and *Anopheles* genera, using canids as natural definitive hosts and humans as incidental hosts [3]. The increasing prevalence of this infection makes it a paradigmatic example of an emerging pathogen [4], influenced by factors such as climate change, the introduction of new competent mosquito species, and human and animal migrations [2, 5, 6]. Inadequate prevention in dogs, the subclinical course of the infection in most of the cases in the animals and low awareness in non-endemic areas also contribute to its spread [4, 7].

To understand the clinical manifestations of this disease, it is useful to briefly consider the parasite’s life cycle. Mosquitoes ingest microfilariae during a blood meal from an infected definitive host, primarily canids [3, 4]. Inside the vector, the microfilariae develop into third-stage (L3) larvae—the obligatory infective stage, which are then transmitted to aberrant human hosts via subsequent mosquito bites [8].

In humans, the larvae typically fail to reach sexual maturity due to immune response [9]. Only a few cases reported the presence of mature adult forms of *Dirofilaria* spp. in humans, producing microfilariae [10, 11].

The clinical presentation is typically dependent on the infecting species [4]. While *D. immitis* causes severe congestive heart failure, mainly in domestic canids [12], in humans it can reach pulmonary arterioles and cause local inflammation, resulting in microinfarcts or granulomas and rarely manifesting clinically [13]. The lesions can also be found as incidentalomas in chest imaging [14]. Few cases described genital localizations of *D. immitis* [15–17].

*Dirofilaria repens*, on the other hand, typically manifests as subcutaneous nodules, often in exposed areas such as the face, neck, and limbs [1, 4]. The nematodes may also reach deeper body areas, such as lymph nodes [18], the abdominal cavity and ovaries [19]. Two cases reported microfilariae crossing the blood-brain barrier in patients with acute neurologic symptoms [20, 21]. Rarer cases of human dirofilariasis in atypical locations have been reported for both species, including the eye [9] and, even less frequently, the genital apparatus [22–24].

This comprehensive review discusses 70 documented cases of human dirofilariasis involving the genital system to consolidate the current understanding of its clinical manifestations and to establish a clear diagnostic and management protocol based on the published literature.

## Materials and Methods

Case reports, case series and review articles describing human dirofilariasis with genital involvement were collected and analyzed. Relevant studies were retrieved through extensive searches in online databases (PubMed, Scopus, Web of Science, Google Scholar) and by manual screening of the reference lists of selected articles. The review included all articles published until October 18, 2025.

Search Strategy.

The search strategy included all relevant results obtained by combining the following keywords: “Dirofilaria”, “Dirofilariasis”, “Dirofilaria repens”, “Dirofilaria immitis”, “Human”, “Genital”, “Scrotum”, “Testis”, “Epididymis”, “Spermatic cord”, “Vulva”, “Vagina”, “Female genitalia”, “Male genitalia”, “Pediatric”, “Child”, “Case report”, and “Review”.

Inclusion and Exclusion Criteria.

Studies were included if they reported clinical cases of human dirofilariasis confirmed by histopathological examination or molecular techniques and in which the anatomical localization of the lesion was specifically indicated in the genital area (male or female).

Cases were excluded if the localization of the lesion was non-genital or if the articles did not provide sufficient clinical or diagnostic details to verify the case characteristics (e.g., lack of species identification or clear anatomical location). Specifically, review articles reporting aggregate case numbers without individual case documentation were not included in the final count.

## Results

This comprehensive review identified and analysed 70 documented cases of genital dirofilariasis establishing the most extensive collection of this rare localization published to date, covering cases from 1990 to 2025. The full data is summarized in Table 1.

**Table 1:** Summary of published detailed cases of genital dirofilariasis

Author/ Year of publication	Country of origin/ infection acquisition	Age	Sex	Clinical Manifestation (Differential diagnosis)	Anatomical location	*Dirofilaria* species	Diagnostic methods	Treatment	Clinical outcome
Kassar et al., 1990 [25]	Tunisia	23y	M	Nodule	Scrotum	*Dirofilaria* species	Histology	Excision	Resolved after excision
Fini et al., 1992 [26]	Italy	52y	M	Orchialgia (Hydrocele)	Epididymis	*D. repens*	Histology	Epididymectomy	NA
Pampiglione et al., 1996 [27]	Greece	3y	M	Nodule	Scrotum	*D. repens*	Histology	Surgery	NA
Auer et al.,1997 [28]	Austria/Italy	35y	M	Orchialgia (Epididymitis)	Epididymis	*D. repens*	Histology, serology + for *D. immitis*	Epididymectomy	Resolved after epididymectomy
Pampiglione et al., 1997 [29]	Italy	52y	M	Swelling (varicocele)	Spermatic cord	*D. repens*	Histology	Excision	Resolved after excision
Pampiglione et al., 1999 [30]	Hungary / Italy	37y	M	Nodule (Tumor/ Testicular Tuberculosis?)	Spermatic cord	*D. repens*	Histology	Orcho-funiculectomy	Resolved after surgery
Ratnatunga et al., 1999 [31]	Sri Lanka	1.5 y	M	Nodule	Scrotum	*D. repens*	Histology	Surgery	NA
“	“	1.5 y	M	Nodule	Scrotum	*D. repens*	Histology	Surgery	NA
Stayerman et al., 1999 [32]	Israel/ Egypt	35y	M	Painless nodule (Fibroma)	Penis	*D. repens*	Histology	Excision	Resolved after excision
Fernando et al., 2000 [33]	Sri Lanka	3y	M	Painless nodule	Scrotum	*D. repens*	Histology	Surgery	NA
Theis et al., 2001 [16]	USA	28y	M	Painful nodule (incarcerated inguinal hernia?)	Spermatic cord	*D. immitis*	Histology, PCR	Orchiectomy	NA
Munichor et al., 2001 [34]	Israel/ Egypt	36y	M	Painless nodule (adenomatoid or malignant tumor?)	Scrotum	*D. conjunctivae*	Histology	Excision	Resolved after excision
Pampiglione et al., 2001 [35]	Italy	NA	M	Nodule	Spermatic cord	*D. repens*	Histology	Surgery	NA
“	Italy	36y	M	Nodule	Epididymis	*D. repens*	Histology	Surgery	NA
“	Italy	44y	M	Nodule	Spermatic cord	*D. repens*	Histology	Surgery	NA
Supriaga et al., 2002 [36]	Russia	12y	M	Funiculitis	Spermatic cord	*D. repens*	Histology	Surgery	NA
Pampiglione et al., 2002 [37]	Italy	71y	M	Nodule (Testicular Tumor?)	Spermatic cord	*D. repens*	Histology	Orcho-funiculectomy	NA
Soussi et al., 2004 [38]	Tunisia	27y	M	Nodule (Abscess)	Scrotum	*D. repens*	Histology	Surgery	Resolved after excision
Angeli et al., 2007 [39]	Italy	28y	M	Swelling (hydrocele?)	Epididymis	*D. repens*	Histology	Surgery	Resolved after excision
Dzamic et al., 2009 [40]	Serbia	NA	M	Swelling (Hydrocele/ Tumor?)	Epididymis	*Dirofilaria* species	Histology	Surgery	Resolved after excision
“	Serbia	40y	M	Swelling (Spermatocoele scrotal tumor?)	Epididymis	*D. repens*	Histology	Surgery	Resolved after excision
“	Serbia	19y	M	Swelling (Scrotal tumor?)	Scrotum- testicle	*D. repens*	Histology	Surgery	Resolved after excision
Fleck et al., 2009 [3]	Germany/ Tunisia	28y	M	Painless nodule (epididymal tumor)	Epididymis	*D. repens*	Histology, serology + for *D. immitis*	Excision	Resolved after excision
Singh et al., 2010 [41]	India	40y	M	Painful swelling	Scrotum	*D. repens*	Histology	Surgery	Resolved after surgery
Di Tonno et al., 2010 [42]	Italy/ Bangladesh	25y	M	Painful nodule (testicular torsion?)	Epididymis	*Dirofilaria* species	Histology	Excision	NA
Joseph et al., 2011 [43]	India	1y	M	Epididymal cyst	Epididymis	*D. repens*	Histology	Excision	NA
D'Amuri et al., 2012 [44]	Italy	45y	M	Swelling (hernia/tumor)	Spermatic cord	*D. repens*	Histology	Orcho-funiculectomy	NA
Leccia et al., 2013 [45]	France	29y	M	Painless nodule	Epididymis	*D. repens*	US, Histology	Nodule resection	NA
“	France	66y	M	Painful nodule	Spermatic cord	*D. repens*	US, Histology	Nodule resection	NA
Kallampallil et al., 2013 [15]	UK	13y	M	Swelling (Epididymitis/ Tumor?)	Epididymis	*D. immitis*	US, Histology	Orchiectomy	Resolved after surgery; testicular prosthesis inserted
Harizanov et al., 2014 [46]	Bulgaria	43y	M	Nodule	Testis	*D. repens*	Histology	Surgery	Resolved after excision
Palicelli et al., 2014 [19]	Italy	52y	F	Ovarian mass (Tumor?)	Ovary	*D. repens*	US, Histology, PCR	Salpingo-oophorectomy	NA
Ilyasov et al., 2015 [47]	Russia	12y	M	Painless nodule	Testis	*D. repens*	US, Histology, PCR	Excision	NA
“	Russia	14y	M	Painless nodule	Testis	*D. repens*	US, Histology, PCR	Excision	NA
“	Russia	7y	M	Painless nodule	Testis	*D. repens*	US, Histology, PCR	Excision	NA
“	Russia	37y	M	Swelling	Testis	*D. repens*	US, Histology, PCR	Excision	NA
Krajina et al., 2015 [48]	Croatia	21y	M	Swelling nodule (Testicular tumor?)	Epididymis	*D. repens*	Histology, serology +	Excision	NA
Bertozzi et al., 2015 [49]	Italy	3y	M	Painful scrotal mass (Acute scrotum/ Torsion?)	Scrotum	*D. repens*	US, Histology, molecular test	Excision	Resolved after excision
Streltsova O.S. et al., 2016 [50]	Russia	39y	M	Nodule	Penis	*D. repens*	Histology	Excision	Resolved after excision
Fedyanina et al., 2016 [51]	Russia	43y	M	Mass (Tumor)	Penis	*Dirofilaria* species	Histology	Surgery	NA
Fuehrer et al., 2016 [52]	Austria/ Namibia	61y	M	Nodule	Epididymis	*D. repens*	Histology, PCR	Surgery	NA
Tumolskaya et al., 2016 [17]	Russia	14 mo	M	Edema, cyanosis (Torsion?)	Scrotum	*D. immitis*	Histology, PCR	Excision	Minor residual edema
Kaftandjiev et al., 2016 [53]	Bulgaria	31y	M	Nodule (Hydrocele?)	Epidipymis	*Dirofilaria* species	Histology	Excision	Resolved after excision
Tripi et al., 2016 [54]	Italy	11 mo	M	Painless nodule (Extra testicular neoplasm?)	Scrotum	*D. repens*	US, Histology	Excision	Resolved after excision
Pigac et al., 2016 [55]	Croatia	3y	M	Painless nodule	Scrotum	*Dirofilaria* species	Histology	Excision	Resolved after excision
Bausch et al., 2017 [56]	Switzerland/India	54y	M	Painful swelling (varicocele/ epididymitis /tumor?)	Epididymis	*D. repens*	US, Histology, PCR -	Excision	NA
Velev et al., 2018 [57]	Bulgaria	11y	M	Painless nodule (epididymal cyst?)	Epididymis	*D. repens*	US, Histology	Excision	Resolved after excision
Sabunas et al., 2019 [58]	Lithuania	79y	M	Nodule	Penis	*D. repens*	Histology, PCR	Surgery	NA
Djakovic et al., 2019 [59]	Croatia	58y	F	Painless Nodule (lymphopathy)	Pubis region	*D. repens*	Histology, PCR	Excision	Resolved after excision
Chandrasena et al., 2019 [60]	Sri Lanka	1y	M	Mass	Scrotum	*D. repens*	US, Histology	Excision	NA
“	Sri Lanka	16y	M	Mass	Scrotum	*D. repens*	Histology	Excision	NA
“	Sri Lanka	46y	M	Mass	Scrotum	*D. repens*	Histology	Excision	NA
Boldis et al., 2020 [61]	Slovakia	46y	M	Nodule (Cyst?)	Epididymis	*D. repens*	US, Histology, PCR	Excision	Resolved after excision
Nagy et al., 2021 [62]	Slovakia/ Turkey	73y	M	Painless nodule (Cyst?)	Epididymis	*D. repens*	US, Histology	Albendazole before orchiectomy	Resolved after orchiectomy
Fassari et al., 2021 [63]	Italy	75y	M	Nodule (Local Infection/ Granuloma?)	Scrotum	*D. repens*	US, Histology	Doxycycline, FNA, Excision, albendazole	Resolved after excision
Pansini et al., 2022 [64]	Italy	11y	M	Painless nodule	Scrotum	*D. repens*	US, Histology	Excision	Resolved after excision
Ugolini et al., 2022 [65]	Italy	13y	M	Swelling	Testis	*D. repens*	US, MRI, Histology, PCR	Excision	Resolved after excision
Sayanthan et al., 2022 [66]	Sri Lanka	4y	M	Painful scrotal mass (Acute scrotum?)	Scrotum	*D. repens*	US, Histology	Excision	Resolved after excision
Rose et al., 2023 [23]	Switzerland	5y	M	Painful scrotal mass (Acute scrotum/ Inguinal hernia/ Epididymitis?)	Spermatic cord	*D. repens*	US, Histology	Excision	Resolved after excision
Kuna et al., 2024 [67]	Poland	21y	M	Painless nodule	Scrotum	*D. repens*	US, PCR	Excision, doxycycline	Resolved after excision
Zulpaite et al., 2024 [24]	Lithuania	42y	M	Painless nodule	Scrotum	*Dirofilaria* species	Histology	Excision	Resolved after excision
Popova et al., 2024 [68]	Bulgaria	59y	M	Painless Nodule (Tumor?)	Scrotum	*D. repens*	Histology	Excision	Resolved after excision
Wijekoon et al., 2024 [69]	Sri Lanka	19 mo	M	Painless swelling (Granuloma?)	Scrotum	*Dirofilaria* species	US, Histology	Emergency excision	NA
“	Sri Lanka	33 mo	M	Painless swelling (Epididimytis?)	Scrotum	*Dirofilaria* species	US, Histology	Emergency excision	NA
“	Sri Lanka	100mo	M	Painful swelling (Epididimytis?)	Scrotum	*Dirofilaria* species	US, Histology	Elective excision	NA
“	Sri Lanka	100mo	M	Painful swelling (Torsion?)	Scrotum	*Dirofilaria* species	US, Histology	Emergency excision	NA
“	Sri Lanka	60 mo	M	Painful swelling (Granuloma?)	Scrotum	*Dirofilaria* species	US, Histology	Emergency excision	NA
“	Sri Lanka	5 8mo	M	Painful swelling (Granuloma?)	Scrotum	*Dirofilaria* species	US, Histology	Emergency excision	NA
Franzò et al., 2025 [22]	Italy/ Srilanka	5y	M	Pain and swelling (Epididymitis/ torsion ?)	Spermatic cord	*D. repens*	US, Histology, PCR	Excision	Resolved after excision
Harch et al., 2025 [70]	Australia/ Sri Lanka	61y	M	Painless nodule	Penis shaft	*Dirofilaria* species Hong Kong genotype	Histology, PCR	Excision	NA

*Abbreviations*: FNA = Fine Needle Aspiration, Y = years, Mo = months, MRI = Magnetic Resonance Imaging, NA = Not available, PCR = Polymerase Chain Reaction, US = Ultrasound

### Analysis of Case Series

The analysis confirms the strong male predominance (over 90% of cases). The demographic analysis revealed an interesting distribution across age groups.

While the condition affects adult males predominantly in European cohorts, 28 patients were aged 16 years or younger, representing 40% of the total cohort. Epidemiologically, a distinct geographic pattern emerged: pediatric cases are mainly concentrated in Sri Lanka, conversely, adult cases are more frequently reported in European countries, specifically in eastern and southern regions.

The parasite showed a particular anatomical tropism in males, primarily affecting the epididymis and the spermatic cord, with clinical presentations often non-specific, as a palpable nodule, and sometimes mimicking acute urological conditions like testicular torsions. The species identification, when performed, shows *D. repens* as the overwhelming causative agent. Three cases were attributed to *D. immitis* [15–17], one to *Dirofilaria* sp. Hong Kong genotype [70] and one case was erroneously reported as *Dirofilaria conjunctivae* [34]. The age range is notably broad, spanning from 11 months [54] to 79 years [58].

## Discussion

Human dirofilariasis is an emerging vector-borne zoonosis of increasing public health importance in Europe, both in areas already known for its presence, (e.g., the Mediterranean basin and eastern European countries like Hungary, Ukraine and Russia) and in newly affected regions (northern Europe) [4]. In contrast to the epidemiological situation in the Old World, where *D. repens* predominates in reported cases of subcutaneous dirofilariasis especially in the Mediterranean area, Sri Lanka and former Soviet Union Republics [1, 9], human cases in North America, and particularly in Florida, are frequently attributed to *Dirofilaria tenuis*, a parasite of raccoons [1]. This species typically presents as subcutaneous or ocular nodules [71]. To date, no cases of genital dirofilariasis have been definitively attributed to *D. tenuis* in the available literature [1].

There are several important factors to consider regarding *Dirofilaria* spread, including inadequate preventative measures in dogs (likely due to the subclinical nature of the infection in canids), and the lack of rapid and reliable diagnostic tests. In addition, other issues regard the low clinical awareness of *D. repens* in non-endemic areas [4], the introduction of new, invasive, competent mosquitoes (e.g., *Aedes* spp.), pet and human migrations [2, 5, 6].

Some authors have explained the rise of cases in specific regions, such as Piemonte in northern Italy, through a combination of environmental and human factors. The presence of rivers, canals, and intensive rice farming creates a humid microclimate. When coupled with broader global climate changes, this provides ideal breeding conditions for competent mosquito vectors in an area already populated by wild fauna that serve as parasite reservoirs [39].

A meta-analysis from the early 2000 indicates that *D. repens* is endemic in all of the Italian peninsula and identified four genital cases in male adults among 60 new cases from 1990 to 1999 [35], while *D. immitis* -related cases are very rare in Italy [72]. Other authors stated that about half of the human dirofilariasis identified in Europe are reported from Italy [11].

The epidemiological threat extends even to isolated areas, as demonstrated by a study reporting human exposure to both *D. immitis* and *D. repens*, with molecular detection of parasites and their endosymbionts, *Wolbachia*, even in small isolated Sicilian islands like Linosa and Lampedusa [73].

Two recent literature reviews have identified 20–28 cases of male genital dirofilariasis in Europe, with fewer than 10 occurring in children [23, 24]. In this review we identified 70 documented genital cases, including 28 in children, three reports of *D. immitis* genital infection [15–17], one of *D. conjunctivae* [34] and the recent *Dirofilaria* spp. Hong Kong genotype [70].

The species *D. conjunctivae* is an historically used term often associated with ocular localization and was already defined as a “questionable” species by major filariasis experts in 1997 [74]. For many years this parasite has been widely considered to be identical to *D. repens* [75] as confirmed by both morphological and molecular data [76]. This classification is critical, as it confirms that the large majority of genital dirofilariasis cases in the Old World are attributable to *D. repens* [1].

The natural definitive hosts of *Dirofilaria* spp. are canines and felids, although a variety of other species like weasels, aquatic mammals, beaver, horses [4] can be infected through the bite of various species of mosquitoes (*Aedes*,* Culex*, and *Anopheles* spp.), acting as vectors and intermediate hosts [3]. Dogs serve as optimal reservoirs for D. repens not only because of their high susceptibility to mosquito bites, but because they can sustain high levels of asymptomatic microfilaremia and often live in close proximity to human habitats [6]. The transmission of *Dirofilaria* spp. requires two main preconditions: the presence of mosquito species capable of transmitting the parasites and the presence of a minimum number of dogs infected with adult helminths that produce microfilariae [9].

Regarding clinical manifestations, in humans, *D. immitis* can reach pulmonary arterioles causing local inflammation, resulting in microinfarcts or granulomas and manifesting clinically with symptoms like chest pain, cough, or hemoptysis [13]. Most of the infections are subclinical in humans because the larvae do not reach the mature form to disseminate in the bloodstream [1]. The lesions can be found as incidentalomas in chest imaging, even mimicking a primary tumor or a metastasis [14]. Without surgical removal, the worms can generally survive also for years without causing harm [3]. In our review we reported three cases describing genital localizations of *D. immitis* [15–17] .

Regarding *D. repens*, usually in a few weeks up to months from the infection, it may stop migrating and form a centimetric subcutaneous nodule in various parts of the body, depending on the site of the bite from the mosquito [23]. The lesions usually consist of a single degenerated worm surrounded by fibrous tissue or also in the context of necrotic tissue with massive inflammation and neo-angiogenesis [10]. The nodule can be erythematous, tender and pruriginous and may be associated, in rare cases, with “cutaneous larva migrans syndrome”, with creeping eruption under the skin [77]. When the worms occasionally reach sexual maturity, microfilariae can migrate through blood vessels to distal sites, but the relation with the immunologic status of the patients in these rare cases is controversial [10]. A case reported microfilariae crossing the blood-brain barrier in a patient with acute neurologic symptoms [21], while another one described eosinophilic meningitis in a patient with intraocular infection caused by *Dirofilaria* spp. Hong Kong genotype [20].

Rarer cases of human dirofilariasis in atypical locations have been reported in deeper body areas, such as lymph nodes [18], the abdominal cavity, ovaries [19], the eye [8, 9] and the genital apparatus [22–24].

The analysis of this review in Table 1 confirms that genital dirofilariasis is a rare condition, predominantly caused by *D. repens*. The most common anatomical locations reported are the epididymis and the spermatic cord, often presenting as a palpable, painful or painless nodule or swelling. We emphasize the importance of excluding this condition considering that the non-specific presentation could lead to misdiagnosis and invasive surgery [16, 17, 19].

The presence of 20 children in this case series highlights the importance of including dirofilariasis in the differential diagnosis even in pediatric settings, especially in people coming from endemic regions like Sri Lanka [75, 78]. The specific anatomical tropism for deep tissues, such as the genital structures remains poorly understood. However, some authors hypothesized that several factors may contribute to the genital localization, including the lower body temperature of the scrotum, which may favor parasite survival; increased patient awareness and consequent self-examination of these body parts, which may lead to earlier detection and a possible tropism of *D. repens* to sexual hormones [4].

Concerning diagnosis, the epidemiological context is crucial for diagnosis of vector-borne diseases [9, 79]. The diagnosis of *D. repens* can be challenging, considering the awareness of the infection [4] and the subclinical course in most cases [1]. We strongly suggest considering the epidemiologic data and anamnesis (travel history, contact with dogs) and a multidisciplinary approach involving radiologists and infectious disease specialists at first, followed by surgeons and expert pathologists when a clinical suspicion is high [22].

**Fig. 1:**
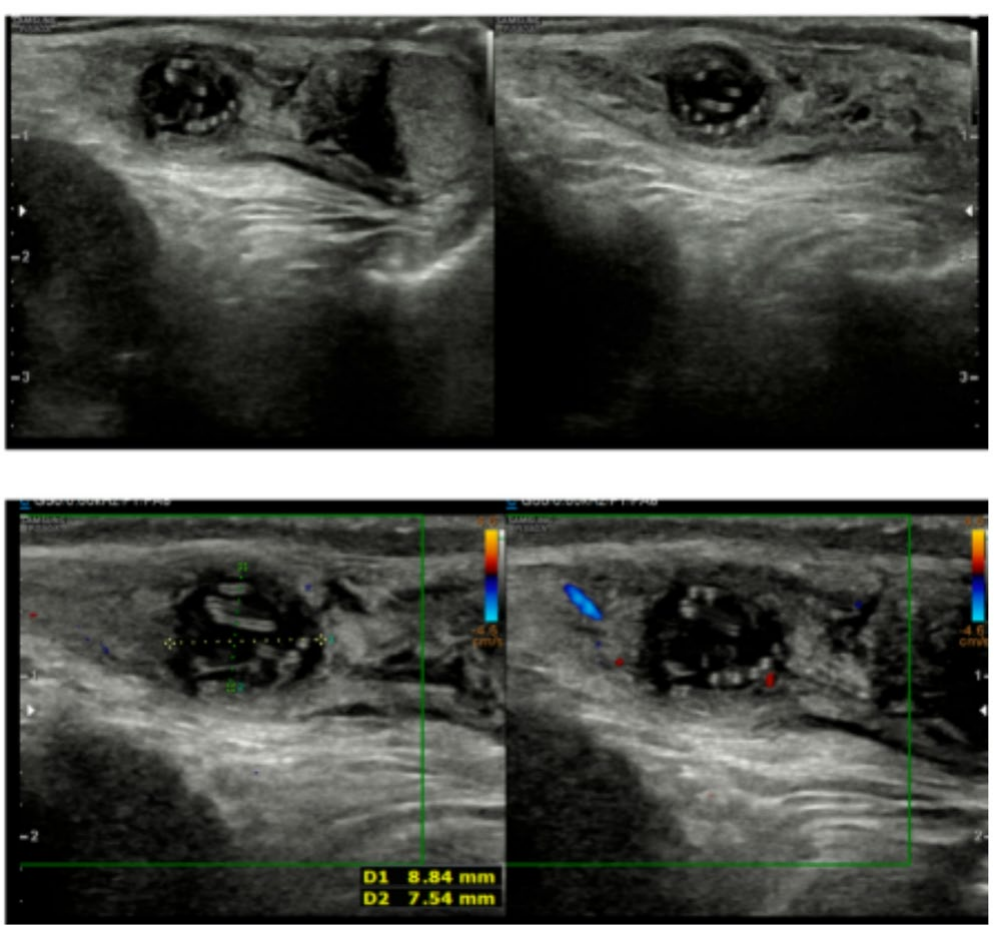
Ultrasound demonstrating a hypoechoic nodule containing linear hyperechogenic lines (worm in-sac-sign)

Ultrasound could be very useful in the initial assessment, particularly given the high rate of pre-operative misdiagnosis, as shown in Table 1, where it is almost routinely used in the last 10 years. The presence of a hypoechoic, encapsulated nodule containing linear, hyperechogenic structures, the “worm-in-sac sign”, is highly characteristic and should prompt suspicion of parasitic infections [22–24]. In our recent case report an initial treatment with non-steroidal anti-inflammatory drugs (NSAID) made the “worm-in-sac sign” visible at the follow-up ultrasound, with reduced inflammation and consequently reduced vascularization on Doppler signal, as shown in Fig. 1 [22].

When the excision of the nodule is performed, the worm can be found in the histopathologic analysis in the context of various inflammatory patterns like granulomatous reaction with neo-angiogenesis or abscess formation, depending on the host reaction. The pathognomonic sign is the presence of a thick multi-layered cuticle with an indented surface as a “gear wheel” – like aspect in transversal section or longitudinal ridges [80]. Some authors contested diagnosis reported in literature, underlining the important role of expert pathologists [81] .

While PCR has been a valuable diagnostic tool since the 1990s [82], some authors have reported discrepancies with histopathology, underscoring the importance of interpreting molecular results in conjunction with clinical and pathological findings [83]. In our recent representative case [22] the definitive diagnosis of *D. repens* was achieved by performing PCR amplification of the ribosomal DNA internal transcribed spacer 2 (rDNA ITS2) region directly on the excised nematode fragment at the closest referring center. Subsequent sequencing of the amplicon and comparison with sequences available in public databases (e.g., NCBI GenBank) confirmed the species with 100% homology, thereby offering the highest level of diagnostic certainty.

Serological tests are not widely available and standardized and so are rarely recommended for the diagnosis of localized human infection, but they can be useful in epidemiological surveys [3, 9, 81]. Significant cross-reactivity with antigens from other filarial worms, such as *Wuchereria* or *Onchocerca*, provide insufficient supportive evidence for definitive diagnosis [83, 84].

Full blood count can sometimes show peripheral hypereosinophilia, rarely in local infections [1, 85].

About treatment, the standard of care for localized genital dirofilariasis is complete surgical excision of the nodule. This approach is diagnostic and, in most cases, completely curative, resulting in resolution of symptoms and no recurrence, as confirmed in Table 1 [1, 4].

Some authors reported treatment with albendazole or ivermectin in combination or not with doxycycline [4, 21]. Ivermectin is sometimes associated with diethylcarbamazine (DEC), unavailable in Italy [84], which, as doxycycline, may play a role in stopping the diffusion of the worm and help the formation of the nodule [78, 85]. Doxycycline can also play a role by targeting the bacterial endosymbiont *Wolbachia* of *Dirofilaria* species [54].

The non-specific clinical presentation of genital dirofilariasis led a few physicians to initiate medical therapy while awaiting a definitive diagnosis, particularly when the suspicion of filariasis was raised by imaging and epidemiology. Three recent case reports illustrate the variable use of antimicrobial therapy in genital dirofilariasis: Nagy et al. [62] reported a case where, upon initial suspicion based on ultrasound findings (the “filarial dance sign” observed after mechanical stimulation), a course of oral albendazole (400 mg/day bis in die for 14 days, followed by a 7-day course after a 10-day break) was recommended by the infectious disease specialist. The patient ultimately underwent orchiectomy, which resulted in the definitive resolution of symptoms. Similarly, Fassari et al. [63] confirmed the resolutive role of surgical excision in a case of testicular tunica infection. Their report noted that initial antibiotic therapy with doxycycline (200 mg daily for one week) resulted in only a mild reduction of the scrotal mass; therefore, surgical removal was required to extract the nematode from the fibrotic nodule. Post-operatively, the treatment was completed with a 3-week course of albendazole (800 mg daily). Lastly, Kuna et al. [67] recently described a case of *D. repens* infection managed with surgical excision followed by a six-week course of oral doxycycline (200 mg daily). Although microfilaremia is rare in humans [10, 11], this pharmacological add-on was prescribed as a precaution, considering the potential occurrence of microfilaremia as described in a case in the authors’ country and the lack of standardized international guidelines. Crucially, all three cases confirmed definitive resolution after surgery, supporting the primary role of excision in localized diseases, as stated in this literature review.

## Conclusions

The findings from this comprehensive review, supported by the aggregated data in Table 1, confirm the rarity and complexity of genital dirofilariasis. The presence of pediatric cases underscores the need for high clinical suspicion, particularly in endemic regions or in patients with relevant travel history, to avoid misdiagnosis and inappropriate interventions like orchiectomy or prolonged antimicrobial therapies. The diagnostic process should progress from initial ultrasound (looking for the “worm-in-sac sign”) to definitive surgical excision and subsequent histopathological and molecular (PCR) confirmation to ensure correct species identification. Complete surgical excision is curative in the absence of microfilaremia, thereby avoiding unnecessary pharmacological treatment and invasive surgery. Genital dirofilariasis should therefore be included in the differential diagnosis of any firm, unexplained scrotal or inguinal mass in patients with an epidemiologic link. A multidisciplinary approach involving infectious disease specialists, radiologists, surgeons, and pathologists is often crucial for the successful management of these cases.

## Data Availability

The authors confirm that the data supporting the findings of this study are included within thearticle and its supplementary materials.
